# Cellular milieu in clear cell renal cell carcinoma

**DOI:** 10.3389/fonc.2022.943583

**Published:** 2022-10-14

**Authors:** Arti M. Raghubar, Matthew J. Roberts, Simon Wood, Helen G. Healy, Andrew J. Kassianos, Andrew J. Mallett

**Affiliations:** ^1^ Kidney Health Service, Royal Brisbane and Women’s Hospital, Herston, QLD, Australia; ^2^ Conjoint Internal Medicine Laboratory, Chemical Pathology, Pathology Queensland, Health Support Queensland, Herston, QLD, Australia; ^3^ Faculty of Medicine, University of Queensland, Brisbane, QLD, Australia; ^4^ Anatomical Pathology, Pathology Queensland, Health Support Queensland, Herston, QLD, Australia; ^5^ Institute for Molecular Bioscience, University of Queensland, Brisbane, QLD, Australia; ^6^ Department of Urology, Royal Brisbane and Women’s Hospital, Brisbane, QLD, Australia; ^7^ Department of Urology, Redcliffe Hospital, Redcliffe, QLD, Australia; ^8^ Centre for Clinical Research, The University of Queensland, Brisbane, QLD, Australia; ^9^ Department of Urology, Princess Alexandra Hospital, Brisbane, QLD, Australia; ^10^ College of Medicine & Dentistry, James Cook University, Townsville, QLD, Australia; ^11^ Department of Renal Medicine, Townsville University Hospital, Townsville, QLD, Australia

**Keywords:** clear cell renal cell carcinoma, proximal tubular epithelial cells, tumor associate macrophages, CD8^+^ T cells, cancer associated fibroblasts

## Abstract

Clear cell renal cell carcinoma (ccRCC) is globally the most prevalent renal cancer. The cells of origin in ccRCC have been identified as proximal tubular epithelial cells (PTEC); however, the transcriptomic pathways resulting in the transition from normal to malignant PTEC state have remained unclear. Immunotherapy targeting checkpoints have revolutionized the management of ccRCC, but a sustained clinical response is achieved in only a minority of ccRCC patients. This indicates that our understanding of the mechanisms involved in the malignant transition and resistance to immune checkpoint therapy in ccRCC is unclear. This review examines recent single-cell transcriptomics studies of ccRCC to clarify the transition of PTEC in ccRCC development, and the immune cell types, states, and interactions that may limit the response to targeted immune therapy, and finally suggests stromal cells as key drivers in recurrent and locally invasive ccRCC. These and future single-cell transcriptomics studies will continue to clarify the cellular milieu in the ccRCC microenvironment, thus defining actional clinical, therapeutic, and prognostic characteristics of ccRCC.

## Introduction

Kidney cancer is the seventh most common adult-onset cancer in Australia (1). At diagnosis, 75% of these kidney cancers will be subtyped as clear cell renal cell carcinoma (ccRCC) with a 5-year survival rate of 50%–69% (2–4). However, if at diagnosis the ccRCC tumor measures greater than 7 cm or has metastasized, then 5-year survival decreases to 10% (3, 5). Clinical outcomes of ccRCC are variable and prediction of survival based on available clinical parameters has been attempted (6). Variability within similar clinical categories occurs, likely due to a combination of limited biomarkers and tumor heterogeneity which hampers more precise prognostication (7). The challenges of poor survival and clinical variation have resulted in numerous detailed cellular profiling studies for ccRCC, providing mechanistic insight for targeted therapeutics. However, gaps persist in our understanding of the complex and variable cell types and states in ccRCC.

Understanding the complex cellular milieu in ccRCC requires knowledge of both individual and integrated cell types and their states. The key cell types in ccRCC are tubular epithelial, immune, and stromal cells that can each attain variable cell states. Individually, these cellular phenotypes have been profiled within ccRCC by various analytical methods. In this review, we summarize the reported individual cellular phenotypes from single-cell transcriptomics studies of ccRCC, to provide an integrated view of key cell types and states that reside in the ccRCC microenvironment.

## Cellular origin of ccRCC—all paths lead to ccRCC

The development of ccRCC is initiated at the gene level. Multiple genomic studies in human ccRCC have revealed a complete or partial biallelic loss in chromosome 3p encoding *VHL* (von Hippel–Lindau tumor suppressor gene) (8). The loss in chromosome 3p has been attributed to faulty chromothripsis, forming micronuclei during mitosis in normal proximal tubular epithelial cells (PTEC) (9, 10). The trigger for the micronuclei formation in normal PTEC has been attributed to their susceptibility to hypoxic microenvironments, a hallmark in ccRCC progression (11, 12). Alteration in *VHL* expression, present in 80%–93% of primary ccRCC cases, forms a self-perpetuating hypoxic PTEC microenvironment (13–17).

### ccRCC development in mouse models

However, singularly this altered *VHL* expression lacks the capacity to induce ccRCC development in mouse models (12, 17–19). Verification of additional genetic alterations in ccRCC was demonstrated in a mouse model study that combined deletion of *Vhl*, transformation-related protein 53 (*Trp53*), and retinoblastoma (*Rb1*) genes to induce ccRCC development (18). In this study, two key aspects of ccRCC were demonstrated. First, the positive staining of malignant cells by proximal tubule protein markers (CD10, AQP1, or NAP12A) confirmed PTEC as the cellular origin of ccRCC (18). Second, the multiple genetic deletions in this mouse model demonstrated a combined genetic variability underlying the development of ccRCC. Similarly, in humans, the development of ccRCC has been reported in PTEC with altered *VHL*, following additional inactivation of polybromo 1 (*PBRM1*), BRCA-associated protein 1 (*BAP1*), and/or SET domain containing 2 (*SETD2*) genes (12, 17, 19).

### ccRCC cellular origin in human studies

Further support for PTEC as the cellular origin of ccRCC has been provided by two human single-cell transcriptomics studies, matching the captured ccRCC PTEC transcriptome to single and/or bulk normal and ccRCC transcriptomes (20, 21). Collectively, these two transcriptomics studies identified the expression of carbonic anhydrase 9 (*CA9*), vascular cell adhesion molecule-1 (*VCAM1*), solute carrier family 17 member 3 (*SLC17A3*), intercellular adhesion molecule 1 (*ICAM1*), integrin subunit beta 8 (*ITGB8*), alpha kinase 2 (*ALPK2*), and vimentin (*VIM*) in ccRCC PTEC (20, 21). Surprisingly, in ccRCC patients the adjacent morphologically normal kidney tissue demonstrated protein marker staining for VCAM1 within CA9-positive PTEC. The VCAM1 and CA9-positive PTEC were termed precursor PTEC and defined as morphologically normal PTEC with *VHL^+/-^* mutation (20). This identification of precursor PTEC in morphologically normal kidney implicates identifiable transcriptomic alteration following genomic alteration. Additionally, this precedes morphological change in ccRCC development and supports a proposed transition from normal to precursor and finally malignant PTEC states.

### An inflamed PTEC state

**Figure 1:**
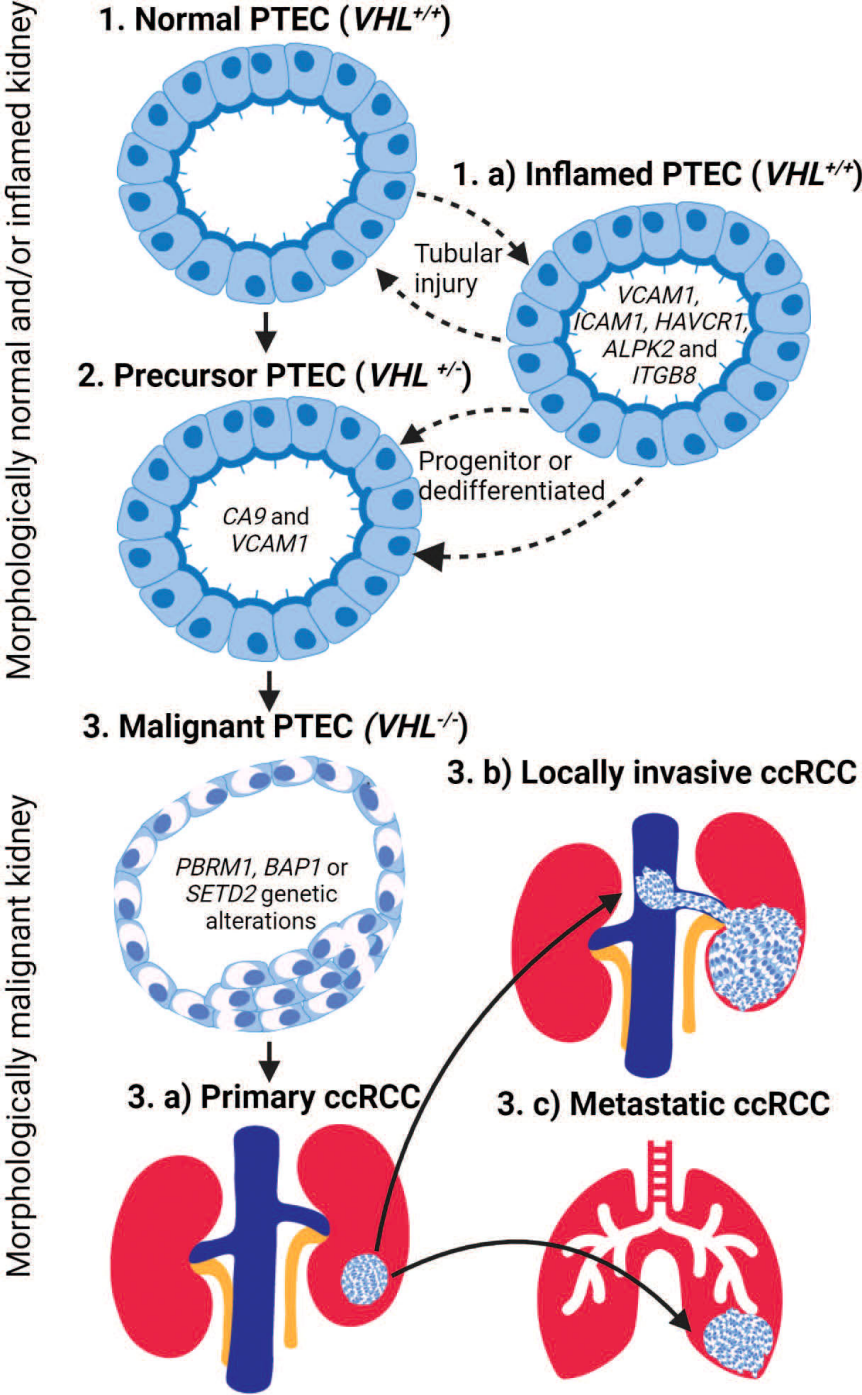
The transition of proximal tubular epithelial cells (PTEC) in the development of ccRCC. PTEC are the cell of origin in ccRCC that under hypoxic conditions transition from normal to malignant state. PTEC transition from normal (Step 1) to precursor *VHL^+/-^* (Step 2) and finally malignant *VHL* ^-/-^ (Step 3) after additional genetic alterations are acquired. Alternatively, under hypoxic conditions, PTEC may transition to an inflamed PTEC *VCAM* (Step 1.a) state due to tubular injury. This inflamed PTEC state is bidirectional until a loss of *VHL* ^+/-^ within the inflamed PTEC is acquired, resulting in the non-reversible transitions to the precursor PTEC (Step 2) state and finally the malignant (Step 3) state after the loss of both *VHL* ^-/-^ and additional genetic alterations are acquired. Once PTEC transition to the malignant state, they can develop primary ccRCC (Step 3.a) lesions within the kidney or locally invasive ccRCC (Step 3.b) lesions that extend into adjacent large vessels and/or metastatic ccRCC (Step 3.c) lesions that spread to distant organs.

Intriguingly, the precursor PTEC expressing VCAM1 and CA9 appear transcriptomically similar to inflamed PTEC with *VCAM1*, but without *CA9* expression. The transcriptomic profile of inflamed PTEC was identified by a multi-omics study performed on normal human kidney tissues (22). These inflamed PTEC are defined with *VCAM1, ICAM1, CD24, CD133*, and *HAVCR1* expression resulting in response to acute and/or chronic tubular injury. Again, this tubular injury is perpetuated by the susceptibility of tubules to hypoxic conditions. Indeed, trajectory inference modeling of the captured PTEC transcriptome revealed a continuum from normal to inflamed PTEC that expanded in tubular injury-related inflammation (21). Since the transcriptomic profile of inflamed PTEC provides the strongest similarity to malignant PTEC, an alternative PTEC transition from normal to inflamed to precursor and finally malignant PTEC state can be proposed in ccRCC development (**Figure 1**).

### Alternative transcriptomics pathway in ccRCC development

The above-mentioned transcriptomic studies validated earlier mouse models by confirming PTEC as the cellular origin of ccRCC in humans (20–22). However, they proposed an alternative inflamed PTEC transcriptomic state under hypoxic injury that perpetuates normal to malignant transition of PTEC in ccRCC development (**Figure 1**). Furthermore, transcriptomic profiling in adjacent normal kidney demonstrates that normal to precursor PTEC transition initiates within morphologically normal kidney. Here, the normal (*VHL^+/+^*) and precursor (*VHL^+/-^*) PTEC transition bidirectionally between these two states. The precursor (*VHL^+/-^*) PTEC transition to irreversible malignant PTEC, if further necessary genetic alterations like *VHL^-/-^* with *PBRM1, BAP1*, and/or *SETD2* mutations are somatically acquired. The proposed alternative transcriptome pathway suggesting normal to inflamed to precursor and finally malignant PTEC transition also initiates within morphologically normal kidney. Here, the normal and inflamed (*VHL^+/+^*) PTEC transition between these two states, as progenitor stem-like PTEC and/or dedifferentiated mature PTEC, to repair the injury that has resulted from transient hypoxic conditions (23, 24). However, during this repair process, mitotic activity increases in the inflamed PTEC, making somatic loss in *VHL^+/-^* plausible and thus allowing irreversible transition to the precursor PTEC state. The final transition to malignant PTEC still requires further necessary ccRCC associated genetic alterations and mutations. While uncertainty remains on whether inflamed PTEC transitioning to the precursor state are progenitor stem-like or dedifferentiated mature (*CD133* and *CD24*) PTEC (22, 24–27), it does raise the possibility that within a subset of ccRCC, the origin may be progenitor stem-like PTEC rather than dedifferentiated mature PTEC (28).

### Locally invasive and metastatic ccRCC

Once PTEC transition to a malignant ccRCC state, they can form (1) a tumor lesion limited to the kidney (2); a locally invasive lesion extending into adjacent medium and/or large vessels; or (3) a metastatic lesion spreading to distant organs. The above-mentioned single-cell transcriptomics profiles are from primary ccRCC lesions. However, a recent single-cell transcriptomics study of ccRCC primary, locally invasive, and adjacent normal tissue identified enhanced extracellular matrix (ECM) remodeling by malignant PTEC in locally invasive lesions (29). This indicates that while locally invasive ccRCC lesions may result from opportunistic extension into vasculature due to proximity, the extending malignant PTEC also require supporting ECM (29, 30). This ECM remodeling can be profiled by the collagen gene markers *COL20A1, COL28A1*, *TGFB1*, *COL6A2, COL1A2*, and *COL4A2.* Similarly, metastatic ccRCC progression has been profiled by 17 metastasis-associated gene (MAG) markers identified in a single-cell transcriptomics study conducted on 121 single cells (31, 32). These single cells were captured from parental metastatic and patient-derived xenografted primary and metastatic ccRCC samples (31, 32). These MAGs include chemokines (*CCL20* and *CXCL1*), and mitochondrial (*MT-ND3, MT-ND4*, and *MT-RNR2*) and cancer (*NDUFA5, NNMT*, *BHLHE41, ALDH1A1*, and *BNIP3*) markers. Expression of these MAG markers is correlated with a higher likelihood of ccRCC recurrence.

In summary, single-cell transcriptomics studies confirm PTEC as the cellular origin of ccRCC. However, the transition from normal to malignant PTEC states may occur *via* several transcriptomics pathways, which are important to define for potential clinical, therapeutic, and prognostic reasons.

## Immune cells in ccRCC—exhausted when things get bad

ccRCCs are defined as immunogenic cancers, which has further been reconfirmed transcriptomically. An immunogenic transcriptome profile initiates with upregulation of gene sets associated with inflammatory cytokines, interferon gamma, and antigen processing on major histocompatibility complex (MHC) by inflamed and malignant PTEC, recruiting immune cells to the ccRCC microenvironment (21, 22, 33). Recruited monocytes enter the kidney tissue and differentiate to macrophages, activating the innate immune response through phagocytosis, exogenous antigen presentation, and immunomodulation (34). The activated innate immune response further recruits T cells, activating the adaptive immune response. In this manner, abundant myeloid and lymphoid cell types and states are recruited to the ccRCC microenvironment, characterizing ccRCC as immunogenic (35–39).

### A dysfunctional immune response in ccRCC

Multiple studies in human ccRCC however, reveal an inverse correlation between abundant immune infiltrate in ccRCC and patient survival, suggesting a dysfunctional immune response (33, 39–43). Understanding this dysfunctional immune response requires an understanding of the infiltrating immune cell types, states, and interactions in the ccRCC microenvironment. Several single-cell transcriptomics and clonal studies have been performed in human ccRCC (21, 33, 42–46), which profile the captured immune cell populations from ccRCC tumor (primary, metastatic, treated, and non-treated), adjacent normal kidney, and/or peripheral blood samples. These provide insight into the transcriptomic profiles of myeloid and lymphoid cell types, states, and their interactions in ccRCC (47–50), with particular emphasis on tumor-associated macrophages (TAMs) and CD8^+^ T cells in the ccRCC microenvironment as both drive the tumor progression and evasion.

### TAM in ccRCC

**Figure 2:**
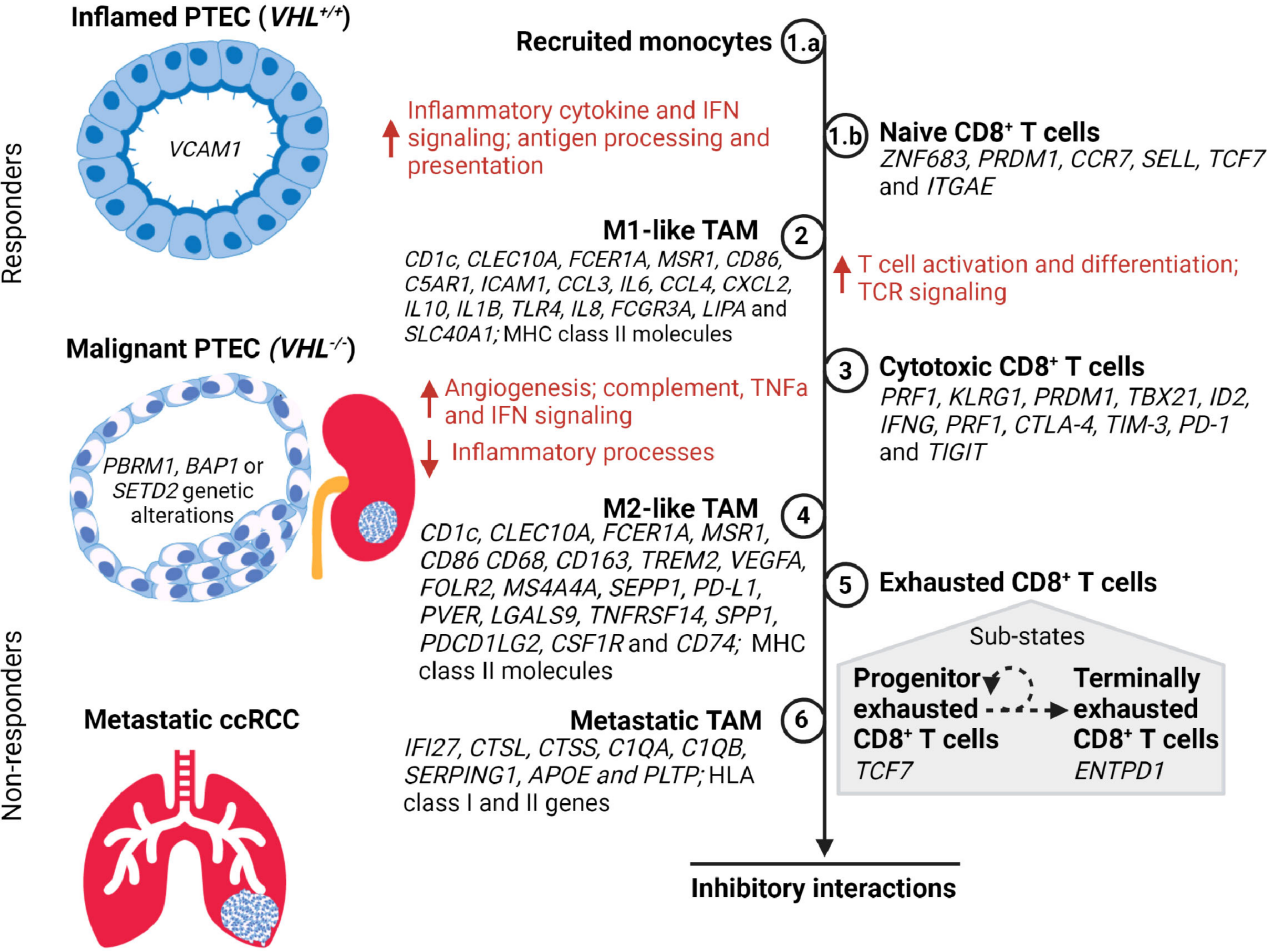
A timeline of tumor-associated macrophage (TAM) and CD8^+^ T cell states during the transition of PTEC in the development of ccRCC. During hypoxic injury, the inflamed PTEC increase the expression of inflammatory cytokine and interferon gamma (IFN) signaling to recruit monocytes (Step 1.a) from the peripheral circulation. These recruited monocytes transition to M1-like TAM states (Step 2) and commence antigen processing and presentation, thus activating the naïve CD8^+^ T cells (Step 1.b) within the kidney to transition to the cytotoxic CD8^+^ T cell state (Step 3). During the malignant PTEC state, the M1-like TAMs increase angiogenesis, complement, tumor necrosis factor alpha (TNFα), and IFN signaling. However, as the ccRCC lesion progresses, the M1-like TAMs transition to the M2-like TAM state (Step 4). The cytotoxic CD8^+^ T cells transition to an exhausted CD8^+^ T cell state (Step 5) composed of a heterogeneous mix of progenitor and terminally exhausted states (51, 52). The subpopulation of exhausted CD8^+^ T cells that exhibit progenitor transcriptome may respond to immune checkpoint therapy until they attain a terminally exhausted CD8^+^ T cell state. In metastatic ccRCC, the M2-like TAM (Step 6) attain metastatic TAM transcriptome profile expressing both the HLA class I and II genes. However, in advancing ccRCC lesions, the M2-like TAMs and exhausted CD8^+^ T cells co-occur and form inhibitory interactions that limit response to immune checkpoint therapy.

Single-cell transcriptomics studies with ccRCC samples have identified synchronous pro-inflammatory M1-like TAMs and anti-inflammatory M2-like TAMs (**Figure 2**). The former are defined by high expression levels of MHC class II molecules and cytokines *IL1B, IL6, IL8*, and *TNF*, while the latter are defined by high expression levels of MHC class II molecules and *CD163, FOLR2, MS4A4A, SEPP1*, and *MSR1* (21, 43). Furthermore, identification of TAM within metastatic ccRCC is defined by high expression of both HLA class I and II genes in conjunction with *IFI27*, *CTSL, CTSS, C1QA, C1QB, SERPING1, APOE*, and *PLTP* (43, 44). These TAM populations within ccRCC demonstrate high plasticity covering a continuum from M1-like to M2-like states; thus, intermediate TAM subpopulations are defined based on HLA-DR or interferon signaling gene expression levels (21, 33, 43, 53, 54). This continuum of TAM states across different stages of ccRCC has been inferred by trajectory analysis to commence from classic/non-classic monocyte to M1-like to M2-like and finally metastatic TAMs across normal, early, locally advanced, and metastatic ccRCC tissue (43). Indeed, a general shift in the TAM states with ccRCC progression is typified by an increase in dysfunctional M2-like TAMs with a simultaneous decrease in M1-like TAMs (43).

### CD8^+^ T cells in ccRCC

Similarly, the transcriptome expression of CD8^+^ T cells in ccRCC samples demonstrates a heterogeneous population and a continuum progressing to terminally exhausted clonotypes (**Figure 2**) (38, 42). Transcriptome expression and/or inferred cell activity has identified naïve, cytotoxic, exhausted, progenitor, and terminally exhausted CD8^+^ T cells (33, 42–45). Transcriptomics and clonotyping profiles in conjunction with inferred pseudotime trajectory analysis of CD8^+^ T cells in ccRCC suggest higher exhausted CD8^+^ T cells with low TCR diversity in advanced and metastatic ccRCC microenvironments compared to those of normal kidney tissues and peripheral blood (39, 42–44). The identification of immune inhibitory markers on CD8^+^ T cells has been concordant with bulk RNA-seq studies demonstrating potential epigenetic reprogramming resulting in exhaustive states *via* TOX2 (39, 55–57). Within the exhausted CD8^+^ T cell population, the identification of progenitor and terminally exhausted subpopulations suggests a spectrum of exhausted states that may transition from progenitor (*TCF7*) to terminally exhausted (*ENTPD1*) state (33, 43, 51, 52).

### Inhibitory interaction between M2-like TAMs and exhausted CD8^+^ T cells

Unlike most CD8^+^ T cells, exhausted CD8^+^ T cells appear to develop many inhibitory interactions with M2-like TAMs, suggesting that ccRCC progression might result from their co-occurrence in advancing ccRCC (43). Due to the loss of spatial information with single-cell transcriptomics, protein marker staining with CD163 (for M2-like TAMs), PD-1, and TIM-3 (for exhausted CD8^+^) has been used to confirm virtual co-localization within the ccRCC microenvironment (43). Further ligand–receptor gene inferencing revealed an increase in immune checkpoint interactions such as *PD-L1*-*PD-1*, *CD80/CD86-CTLA4*, *NECTIN2/PVR-TIGIT, LGALS9-TIM-3*, *TNFRSF14-BTLA*, and *SPP1-CD44* between M2-like TAMs and exhausted CD8^+^ T cells, in advanced ccRCC (43). Inversely, the identification of *CSF1* and *MIF* ligands on exhausted CD8^+^ T cells suggests M2-like polarization *via* interactions with *CSF1R* and *CD74* receptors on TAMs. These inferred ligand–receptor interactions between M2-like TAMs and exhausted CD8^+^ T cells suggest that their inhibitory interaction increases as ccRCC progresses.

### M2-CD8 exhaustion gene signature correlates with worse survival in ccRCC

Therefore, both TAMs and CD8^+^ T cells transition to an anti-inflammatory and exhaustive state within the tumor microenvironment as ccRCC progresses (**Figure 2**). These dysfunctional M2-like TAMs and exhausted CD8^+^ T cells additionally form inhibitory interactions that further perpetuate a dysfunctional immune response. To confirm immune dysfunction resulting from the inferred inhibitory interactions, there has been further investigation of the generated M2-CD8 exhaustion gene signature. First, the expression of this M2-CD8 exhaustion signature was confirmed by mass cytometry (54) and the Cancer Genome Atlas (TCGA) (58) ccRCC datasets to be present in advanced ccRCC. Next, the effect that M2-CD8 exhaustion has on treatment outcomes was investigated in advanced and/or metastatic ccRCC patients treated with either PD-1 blockade or mTOR inhibition (43, 59). This showed no association between response or progression-free survival with the expression of M2-CD8 exhaustion signature with either treatment. In fact, increased M2-CD8 exhaustion signature in TCGA and treatment datasets correlated with worse overall survival in ccRCC patients (43). This suggests that M2-like TAMs and exhausted CD8^+^ T cells may not respond to PD-1 blockade in ccRCC as is clinically expected.

### A subset of progenitor exhausted CD8^+^ T cells in ccRCC

Clinical trial data suggest that ccRCC does otherwise respond to immune checkpoint blockade (ICB), like PD-1 inhibitor (60). The function of ICB is to block inhibitory signals that limit immune cell activation, thus allowing tumor reactive immune cells to overcome this pro-tumor regulatory mechanism and initiate an effective anti-tumor immune response (61). Indeed, other studies demonstrate that exhausted CD8^+^ T cells include a subset of progenitor exhausted CD8^+^ T cells (*TCF7*) within the tumor microenvironment that respond to PD-1 blockade and then transition to a terminal exhausted (*ENTPD1*) state (**Figure 2**) (43, 51, 52, 62–66). Further investigation of ccRCC transcriptomics data has identified this terminal exhausted subset within the progenitor exhausted CD8^+^ T cell population with *TNFRSF9 (*or *4-1BB* ^Low^) and upregulated *GZMA* and *FASLG*, confirming the presence of progenitor exhausted CD8^+^ T cells (33). Based on this, it can be concluded that effective response to immune checkpoint therapy in advanced and metastatic ccRCC requires an absolute or relative absence of dysfunctional M2-like TAMs and exhausted CD8^+^ T cells, or the presence of progenitor exhausted CD8^+^ T cells. Resistance to immune checkpoint therapy in ccRCC can additionally be attributed to failed reversal or reinvigoration of dysfunctional M2-like TAMs and exhausted CD8^+^ T cells, as has been suggested in other cancers (42–45, 56, 57, 67–71). Therefore, it is controversial whether additional checkpoint therapies, targeting additional immune checkpoints, will confer clinical benefit to ccRCC patients with an immune profile composed of M2-like TAMs and terminally exhausted CD8^+^ T cell states. Unlike other solid malignancies, tumor mutation burden and PD-L1 status in ccRCC are not predictive indicators of immune checkpoint therapy outcome (46), suggesting that dysfunctional immune responses associated with infiltrating immune cell types and states may be better predictors of clinical response and therapeutic resistance to immune checkpoint therapy in ccRCC.

## Stromal cells in the ccRCC microenvironment—an elusive tumor driver

In ccRCC, the biallelic loss of *VHL* alleles in malignant PTEC activates and stabilizes the hypoxia-inducible factors (HIFs), further supporting the transcription and secretion of HIF target genes, like vascular endothelial growth factor (*VEGF*) (21). This *VEGF* upregulation in the ccRCC microenvironment supports proangiogenic and immunosuppressive processes. The above-mentioned single-cell transcriptomics studies have inferred angiogenic activity with the secretion of *VEGFA* ligand by malignant PTEC and macrophages, which interact with the VEGF-signaling receptors on endothelial cells (*KDR, FLT1*, *NRP2, NRP1*, and *ACKR1* **),** macrophages (*NRP2* and *NRP1*), and fibroblasts (*NRP1*) (20, 21, 39, 45). Furthermore, angiogenic and proliferative activity in ccRCC has been inferred *via PGF* and *EFNA1* ligands secreted by malignant PTEC, which interact with the receptors on endothelial cells, TAMs, and fibroblasts (39). These transcriptome profiles support the well-known proangiogenic activities within the ccRCC microenvironment, morphologically characterized as a highly vascular tumor with favorable response to antiangiogenic treatments (72).

### Cancer-associated fibroblasts in recurrent and locally invasive ccRCC

**Figure 3:**
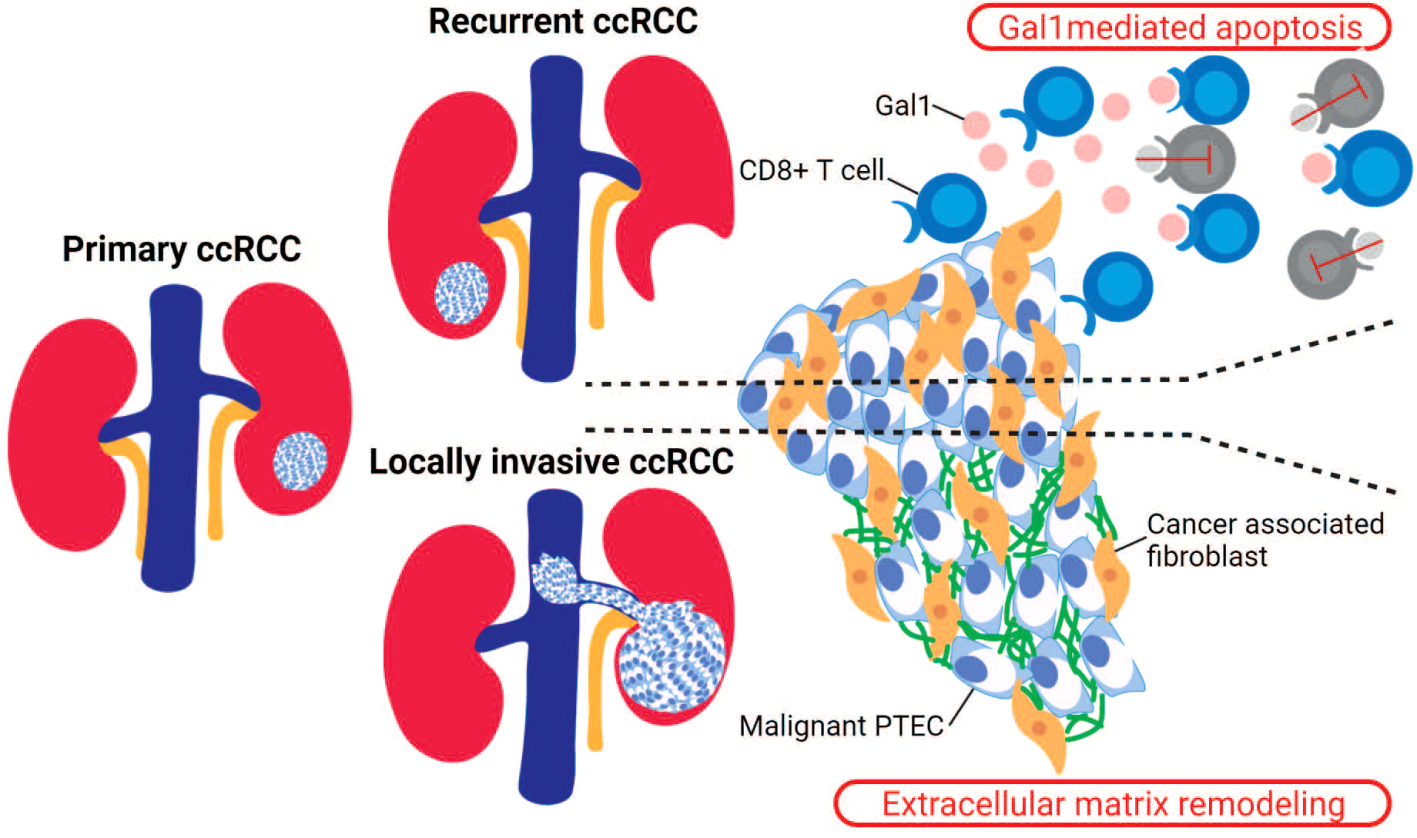
Stromal cells in recurrent and locally invasive ccRCC. Cancer-associated fibroblasts (CAF) are stromal cells that have been identified as drivers in the recurrent and locally invasive ccRCC microenvironment. In recurrent ccRCC, the infiltrating CAF secrete Gal1 that binds to the activated CD8^+^ T cells and thus mediating T cell apoptosis. In locally invasive ccRCC, the infiltrating CAF increase the expression of genes within the epithelial–mesenchymal transition (EMT) pathway, leading to an increased extracellular matrix remodeling within the invasive ccRCC lesion.

However, like immune checkpoint therapies, antiangiogenic treatments fail to maintain a sustained clinical response in ccRCC patients. Thus, some focus and attention has turned to non-malignant and non-immune stromal cells. These include cancer-associated fibroblasts (CAF) within the ccRCC microenvironment due to their possible immunosuppressive functions. The recruitment of CAF within the ccRCC microenvironment is proposed to occur *via* interactions with malignant PTEC that upregulate *COL20A1, COL28A1*, and *TGFB1* (29). These infiltrating CAF are able to reduce CD8^+^ T cell infiltration within ccRCC microenvironments, particularly within recurrent ccRCC as identified by a recent single-cell transcriptomics study (**Figure 3**) (73). In this study, the immunosuppressive behavior mediated by CAF was attributed to the secretion of Galectin-1 (Gal1), which was noted within the captured transcriptome by the substantial expression of *LGALS1*. Gal1, a well-known immunosuppressor within various tumor microenvironments, mediates apoptosis of cytotoxic CD8^+^ T cells. This apoptotic activity of CAF was demonstrated within both *in vitro* and *in vivo* Gal1 knockdown models. Furthermore, the immunosuppressive nature of CAF was confirmed by reduced progression-free survival in ccRCC patients whose malignancies were observed to have high CAF infiltration and who had received immune checkpoint therapy (73). In addition to these immunosuppressive properties within the tumor microenvironments, the secreted Gal1 by CAF has been reported to promote epithelial–mesenchymal transition (EMT) in gastric cancer. Interestingly, CAF mediated EMT has been proposed within locally invasive ccRCC that rapidly extends into surrounding large vessels (**Figure 3**). Here, a single-cell transcriptomics study in locally invasive ccRCC identified CAF-mediated extracellular matrix remodeling by the increased gene signature for the EMT pathway (29). Therefore, in both recurrent and locally invasive ccRCC, CAF infiltrate should be considered as an additional key cell type driving tumor progression and immunosuppression (29, 73).

## Conclusion

ccRCCs are characterized as hypoxic, immunogenic, and angiogenic tumors. An understanding of ccRCC requires investigation of all these characteristics not only within tumor cells but also in immune and stromal cells that infiltrate the ccRCC microenvironment. Recent application of single-cell transcriptomics within the ccRCC tumor (primary, metastatic, treated, and non-treated), adjacent normal kidney, and/or peripheral blood samples has expanded our understanding of the divergent cell types and states of ccRCC. The identification of inflamed PTEC poses the possibility of an alternative transcriptomic pathway in the development of ccRCC. There is growing evidence suggesting a dysfunctional interaction between M2-like TAMs and exhausted CD8^+^ T cells and/or the lack of progenitor exhausted CD8^+^ T cells in advanced and metastatic ccRCC might play a major role in resistance to available immune checkpoint therapies. Recent identification of immunosuppressive and extracellular matrix remodeling activities by CAF suggests stromal cells as additional elusive drivers in recurrent and locally invasive ccRCC. Therefore, a complete account of PTEC, immune and stromal cell types and states within the ccRCC microenvironment is shedding light on tumor progression and evasion in early, local, and metastatic ccRCC and informing future clinical management, therapeutics, and prognostics.

## Author contributions

AR, MR, SW, and AM conceived the review. AR, MR, and AK undertook the literature research and write-up. MR, SW, HH, AK, and AM reviewed the manuscript. All authors contributed to the article and approved the submitted version.

## Funding

AR is supported by an Australian Government Research Training Program (RTP) Scholarship and funding from Pathology Queensland—Study, Education and Research Committee.

## Acknowledgments

Parts of all figures were created with Biorender.com.

## Conflict of interest

The authors declare that the research was conducted in the absence of any commercial or financial relationships that could be construed as a potential conflict of interest.

## Publisher’s note

All claims expressed in this article are solely those of the authors and do not necessarily represent those of their affiliated organizations, or those of the publisher, the editors and the reviewers. Any product that may be evaluated in this article, or claim that may be made by its manufacturer, is not guaranteed or endorsed by the publisher.
